# Morphinofobia: the situation among the general population and health care professionals in North-Eastern Portugal

**DOI:** 10.1186/1472-684X-9-15

**Published:** 2010-06-22

**Authors:** Henk Verloo, Emmanuel K Mpinga, Maria Ferreira, Charles-Henri Rapin, Philippe Chastonay

**Affiliations:** 1 Geneva Altitude Clinic, Montana, Switzerland; 2 Department of Community health and medicine, Faculty of medicine, University of Geneva, Geneva, Switzerland; 3 Formerly RN in the hospital Castèlo Branco, Beira Interior, Portugal and responsible of chirurgical ward hospital of Siders, RSV, Switzerland; 4 Faculty of Medicine, University of Geneva, Geneva, Switzerland; 5 Faculty of medicine, University of Geneva, Geneva, Switzerland and Director of the Department Age, Health and Society, University Institute Kurt Bösch, Sion, Switzerland

## Abstract

**Background:**

Morphinofobia among the general population (GP) and among health care professionals (HP) is not without danger for the patients: it may lead to the inappropriate management of debilitating pain. The aim of our study was to explore among GP and HP the representation and attitudes concerning the use of morphine in health care.

**Methods:**

A cross-sectional study was done among 412 HP (physicians and nurses) of the 4 hospitals and 10 community health centers of Beira Interior (Portugal)and among 193 persons of the GP randomly selected in public places. Opinions were collected through a translated self-administered questionnaire.

**Results:**

A significant difference of opinion exists among GP and HP about the use of morphine. The word morphine first suggests drug to GP (36,2%) and analgesia to HP (32,9%.). The reasons for not using morphine most frequently cited are: for GP morphine use means advanced disease (56%), risk of addiction (50%), legal requirements (49,7%); for HP it means legal risks (56,3%) and adverse side effects of morphine such as somnolence - sedation (30,5%) The socio-demographic situation was correlated with the opinions about the use of morphine.

**Conclusions:**

False beliefs about the use of morphine exist among the studied groups. There seems to be a need for developing information campaigns on pain management and the use of morphine targeting. Better training and more information of HP might also be needed.

## Background

During the last decades, considerable progress has been recorded in the knowledge of the action and the use of opiates in pain management [1-3]. In spite of this progress, the pain prevalence in general populations and health care institutions remains high, varying from 20 to 80% depending on the region or the country [4-7].

Why has this knowledge not been applied by HP and did not have the expected effects among the GP? Do morphine frighten so much the GP and HP becoming a public health problem?

Morphinofobia can be defined as a set of false beliefs concerning the negative effects of morphine in the management of pain [8], an inappropriate attitude of professionals in the pain management due to a lack of knowledge [9], a philosophical opposition to the prescription and the use of morphine in pain management [10].

Morphinofobia seems widespread and caused by ignorance, prejudices, false beliefs, economic marketing strategies and limitations in the availability of morphine [8,10-16].

In 1960, the studies of Robins *et al. *[17] and Abeles *et al. *[18] reported false beliefs of health professionals in the use of morphine: it was related to fears of addiction and abuse, to limited information on legal aspects, a lack of knowledge about the use of opiates by health professionals (physicians) and users (patients) and to the negative image of morphine in general. Similar observations have been reported in a recent study by Zacny *et al. *[19]: morphine was often associated with advanced disease, imminent death, illicit drug addiction, euthanasia, potential risks of abuse, excessive sedation and fear of pursuit by authorities [5,7,10,14,20-25]. Few studies compared attitudes and perceptions related to the use of morphine as an analgesic among GP and HP in a given region.

Musi *et al. *[26] studied the myths of morphine in the Valley of Aosta in Northern Italy interrogating 380 health professionals and the general population about their fears in using morphine. They showed that despite the availability of morphine, its low costs and its efficacy, the prescription and the acceptance of opioïdes, and more specifically of morphine, in health care institutions was low.

Our study aims to compare morphinofobia among the general population (GP) and health professionals (physicians and nurses) (HP) in a country where the consumption of morphine was multiplied by 4 over the last decade [27], though its prescription is tightly regulated by public health authorities [28].

## Methods

The survey was carried out between August and November 2005 using two structured questionnaires, developed based on the model of Musi *et al. *[26]. One of the authors (MF) translated the questionnaires in Portuguese and later conducted the survey. After a translation check by two Portuguese health professionals the questionnaires were pilot-tested among 5 GP and 5 HP in the province of Beira Interior.

### Data Collection

The GP was recruited randomly on a given day in two shopping centres, three urban restaurants, the weekly marketplace and at the railroad station of Guarda. The participation criteria were: at least 18 years old, able to answer the questionnaire and living in the region of Beira Interior.

As to the GP, a questionnaire was addressed to 800 HP (nurses and physicians) employed at four hospitals of Beira Interior (hospitals of Cova da Beira, Fundão, Guarda and Castélo Branco) and ten community care centres (Belmonte, Castélo Branco, Covilhã, Fundão, Idanha-A_Nova, Oleiros, Penamacor, Provença-a- Nova, Sertã, Vila Velha of Rodão) with the agreement of the regional Department of Health of Beira Interior.

The HP were working in internal medicine, general surgery, paediatrics, oncology, orthopaedics, emergency and community home care. The participation criteria were: be employed in one of the hospitals or community home care centres for at least a year and having a completed training as a nurse or as a physician.

The questionnaires were distributed via the management of the institutions and answers were returned by the same channel under ceiled envelope. The inquiry was anonymous. The study was approved by the Research Ethics Committee of the University Institute of Kurt Bösch Sion, Switzerland.

### Research Instrument

For each group, we developed a specific questionnaire based on the study of Musi *et al. *[26].

The first part of each questionnaire addresses socio-demographic data. The second part of the questionnaire addresses the perception people have of "morphine", its efficacy and its side effects. The questionnaire also explores the attitudes concerning the use of morphine and its acceptance. A judgment scale of 5 levels, ranging from "completely disagree" to "completely agree" was used.

Data was analyzed with « Khi ^2^of Pearson », « t-test for matched samples » and « correlation of Pearson » using the software program SPSS version 15.0. Significant differences (p-value) between categories or groups of variables were defined at 95%.

## Results

The sampling among GP and HP yielded a total of 606 respondents.

### Profile of Respondents

#### General population (GP)

194 persons of GP answered the questionnaire. One questionnaire was discarded. About six out of ten respondents were women. Age range was between 18 and 80 years. About 20% of the respondents had not been to school and about 30% had only attended primary school. Almost half of the interviewed people lived in urban areas. A vast majority of respondents (87%) were Catholics (Table 1). The demographic profile of our so-called GP roughly corresponds to the demographic distribution of the population of the region Beira Interior, except for gender, yet the studied sample must be considered as an opportunistic sample.

**Table 1:** Characteristics of the GP and HP

Variables	GP n (%)	HP n (%)	P- Value
Sex of respondents (GP n = 176-H P n = 412)			
Male	82 (42)	108 (26,2)	0,000*
Female	111 (58)	304 (73,8)	

Age of respondents (GP n = 174 -H P n = 396)			
18-25 years	27 (15,5)	82(19,9)	
26-65 years	102 (58,6)	313(76,0)	0,000*
>65	44 (25,9)	1 (0,2)	
No response	3	16	

Level of training (GP n = 176 -H P n = 412)			
Without schooling	36 (20,5)		
Only primary	50 (28,4)		
Secondary school	46 (26,1)		0,000*
High school	20 (11,4)	366 (88,8)	
High school	20 (11,4)	366 (88,8)	
University	24 (13,6)	46 (11,2)	
No response	18	-	

Place of living (GP n = 176 - HP n = 409)			
Rural	78 (44,3)	123 (24,4)	
Urban	98 (55,6)	284 (75,6)	0,001*
Other	-	5	
No response	17	-	

Religion (GP n = 175 - HP n = 408)			
Catholic	152 (86,9)	385 (93,4)	
Jewish	1 (0,6)	2 (0,5)	
Jehovah	22 (12,6)	2 (0,5)	0,000*
Protestant	-	7 (1,7)	
Other		16 (3,9)	
No response	18	-	

* Significant difference P-value ≤ 0.05

#### Health Professionals (HP)

Of 700 questionnaires addressed to nurses and 100 to physicians, 412 were returned. The sample included 366 nurses (89%) and 46 physicians (11%). The participation rate of nurses was 52,3% the physicians' 46,0%. On the average the participation rate was 51,5%. Three quarters of the respondents were women. The average age was 35,5 years. About 70% of the respondents lived in semi-urban areas. The majority of the HP (93%) were Catholics (Table 1). The sample roughly represents the doctors/nurses rate in health care in Portugal. Non-respondents were not specifically characterized in our study, but were globally not different from the respondents (same sex ratio, age distribution, religion, and years of professional experience).

### The Myths of Morphine

#### Perception of the word « Morphine »

Significant differences exist between GP and HP in their perception of the word "morphine". For HP the word "morphine" first stands for « *analgesic *» (32,9% ), whereas for GP it first means «*drugs*» (36,2%). Other differences exist between GP and HP, such as «*medication*» (15,9% versus 5,2%,), "*sedation*- *somnolence*» (9,1% - 0,5%), «*cancer*» (0% versus 14,9%), «*dependency*» (0% versus 1,8%,) «*opiate*» (6,9% versus 15,9%), «*relief*» (0% versus 16,4%). Some similarities also exist, such as «*pain-suffering*» (GP 1,7% versus HP4,1%) and «*end of life *- *death*» ( GP 2,3% versus HP 2,8%). One third of GP *« does not know *» what morphine stands for. The results are summarized in Table 2.

**Table 2:** Perception of the word « morphine » among the GP and HP (only one answer possible)

GENERAL POPULATION	n = 176 (%)	HEALTH PROFESSIONELS	n = 412 (%)	P-VALUE
**Drug**	**63 (35,7)**	**Analgesic**	**128 (32,9)**	

**Don't know**	**57 (32,3)**	**Relief**	**64 (16,5)**	

**Medication**	**28 (15,9)**	**Opiate**	**62 (15,9)**	

**Sedation- somnolence**	**16 (9,1)**	**Cancer**	**58 (14,9)**	

**Opiate**	**12 (6,8)**	**Drug**	**43 (11,1)**	

**Analgesic**	**11 (6.2)**	**Medication**	**20 (5,2)**	0,000 *

**End-of-Life - Death**	**4 (2,3)**	**Pain-Suffering**	**16 (4,1)**	

**Pain- Suffering**	**3 (1,7)**	**End-of-Live - Death**	**11 (2,8)**	

		**Dependency**	**7 (1,8)**	

		**Sedation-Somnolence**	**2 (0,5)**	

		**No response**	**24**	

* Significant difference p- value ≤ 0.05

#### Opinions about the use of morphine as an analgesic

The opinions among GP and HP concerning the use of morphine as an analgesic appear in Table 3.

**Table 3:** Opinions about the use of morphine as an analgesic among the GP and HP

OPINIONS ABOUT USE OF MORPHINE	GP AGREEMENT (%)	HP AGREEMENT (%)	% DIFFERENCE GP - HP	P-Value
**It means that it is serious **(GP n = 171; HP n = 399)	96 (56,1)	37 (9,3)	46,8	0,000*

**There is a risk to develop dependency **(GP n = 171; HP n = 405)	70 (41.0)	6 (1,5)	39,5	0,000*

**There is a risk of delirium or euphoria **(GP n = 171; HP n = 405)	68 (39.8)	18 (4,4)	35,4	0,000*

**It does diminish the surviving period **(GP n = 171; HP n = 407)	61 (35,7)	13 (3,2)	32,5	0,002*

**There is a risk of somnolence or sedation **(PG n = 170; PS n = 403)	74 (43,5)	123 (30,5)	13,0	0,000*

**It can lead to increasing doses **(GP n = 170; HP n = 403)	85 (50,0)	105 (26,1)	23,9	0,000*

**It is a sign of limited life expectancy **(GP n = 171 - HP n = 409)	47 (27,5)	4 (1,0)	26,5	0,000*

**It increases legal risks in relation to other medication **(GP n = 171; HP n = 407)	85 (49,7)	229 (56,3)	-6,6	0,149

**There is a risk of discrimination **(PG n = 169; PS n = 397)	33 (19,5)	10 (2,5)	17,0	0,000*

* Significant difference P value ≤ 0.05

GP shows more false beliefs than HP concerning the use of morphine as an analgesic. The largest difference exists for «*it means that it is serious*» (46,8%; p = 0,000), the smallest for «*there is a risk of somnolence or sedation*» (13%; p = 0,002.)

### Relationship between socio- demographic features and the perceptions of the use of morphine as an analgesic

Table 4 presents a matrix of the correlation coefficients of Pearson between socio-demographic features and opinions on the use of morphine as an analgesic.

**Table 4:** Matrix of the correlation coefficients (Pearson) between the socio-demographics characteristics and the opinions about the use of morphine as an analgesic

OPINIONS ABOUT USE OF MORPHINE	SEX	AGE	LEVEL OF TRAINING	PLACE OF LIVING
**Its means that it is serious - Correlation coefficient**	-0.134**	0.403**	-0.375**	-0.134**
Sig. (bil.)	0.001	0.000	0.000	0.001
N	570	554	570	568

**Risks of developing dependency Correlation coefficient**	-0.094*	0.481**	-0.545**	-0.119**
Sig. (bil.)	0.024	0.000	0.000	0.004
N	575	558	575	573

**Risks of delirium or euphoria - Correlation coefficient**	-0.041	0.469**	-0.467**	-0.053
Sig. (bil.)	0.330	0.000	0.000	0.207
N	576	559	576	574

**Diminish surviving period - Correlation coefficient**	-0.051	0.514**	-0.583**	-0.145**
Sig. (bil.)	0.223	0.000	0.000	0.000
N	578	561	578	576

**Risks of somnolence or sedation - Correlation coefficient**	-0.110**	0.254**	-0.192**	0.025
Sig. (bil)	0.008	0.000	0.000	0.547
N	577	560	577	575

**Risks of increasing doses - Correlation coefficient**	-0.058	0.265**	-0.241**	-0.032
Sig. (bil.)	0.166	0.000	0.000	0.441
N	573	556	573	571

**Limited life expectancy - Correlation coefficient**	-0.125**	0.474**	-0.488**	-0.147**
Sig. (bil.)	0.003	0.000	0.000	0.000
N	580	563	580	578

**Legal constrains- Correlation coefficient**	0.025	0.129**	-0.106*	-0.034
Sig. (bil.)	0.543	0.002	0.011	0.412
N	578	561	578	576

**Risks of discrimination - Correlation coefficient**	-0.096*	0.278**	-0.517**	-0.169**
Sig. (bil.)	0.023	0.000	0.000	0.000
N	566	549	566	564

* Significant correlation on 0,05 level (bilateral)	** significant correlation on 0,01 level (bilateral)

Data analysis shows an absence of a significant relationship between the **sex **of the respondents and the questions «*risks of delirium*» (r = 0.041; p = 0,330), «*diminish the surviving period*» (r = 0,051; p = 0,223), «*risks of increasing doses*» (r = 0,058; p = 0,166) and «*the legal risks*» (r = 0,025; p = 0,543). A weak negative relationship was seen between sex and the expressions «*it means that it is serious*» (r = 0,134; p = 0,001), «*risks of dependency*» (r = 0,094; p = 0,024), «*risks of somnolence*» (r = 0,110; p = 0,008) «*limited life expectancy*» (r = 0,125; p = 0,003) and «*risks of discrimination*» (r = 0,096; p = 0,023). Men are less prone to consider and use morphine as an analgesic than women.

A positive weak relationship was observed between the **age **of the respondents and the perceptions of the use of morphine. The older the respondents, the more false beliefs exist about the use of morphine.

A weak negative relationship between ***level of training ***of the respondents and the variable «*legal risks*» (r = 0,106; p = 0,011) was observed. A weak positive relationship was observed between ***place of living ***and the expressions «*it means that it is serious*» (r = 0,134; p = 0,001), «*risks of dependency*» (r = 0,119; p = 0,004), «*diminish surviving period*» (r = 0,145; p = 0,000), «*limited life expectancy*» (r = 0,147; p = 0,000) and «*risk of discrimination*» (r = 0,169; p = 0,000). No relationship was noticed with the variables *«risk of delirium» (r = 0,053; p = 0,207), «risks of somnolence» (r = 0,025; p = 0,547), «risks of accustom» (r *= 0,032; p = 0,441) and *"legal risks» *(r = 0,034; p = 0,412).

## Discussion

This study compared the use of morphine as perceived by GP and HP in the region of Beira Interior in North-Eastern Portugal. There are differences of perception but also common fears. It might well induce some reluctance regarding the use of morphine. This in turn might influence negatively patient care in general and pain management more specifically.

Most studies reporting "false beliefs" regarding the use of morphine in pain management focus either on specific ethnics groups or on health professionals [13,4,29-31]. Studies comparing GP and HP in this field are few. One done by Musi *et al. *[26] in Northern Italy confirms our results. These authors mention than 39% of GP primarily associate the word morphine with «drugs» and «the risks of somnolence, dependency and the seriousness of the clinical situation». Other studies also support our observations. Weisse *et al. *[31] report that the physicians' attitude in prescribing analgesics for pain management varies according sex and ethnic group. Bernades [32] reports a difference in perception of pain according to sex. Riley *et al. *[29,33] and Robinson *et al. *[30] show a significant difference in chronic pain management according to age.

In our study morphinofobia among HP seems related to false beliefs on side effects of morphine, risks of addiction and legal constraints in the prescription of morphine. Yet the word morphine is principally associated with the notion of analgesia. Musi *et al. *[26] reports in his study among HP that the word morphine is associated first with « pain » followed by «analgesia, drug, cancer, death and sedation» which does not differ much from our observations. Other authors report similar data to ours. Seddon *et al. *[34] mention clearly that the use of morphine in pain management is strongly influenced by the society's perceptions, especially as far as addiction and the legal constrains go. The recent Italian study of Bandieri et al. [13] analysed the consumption of opioids between 2000 and 2008 and showed an increase in the use of opioids in general, but a decrease use of oral morphine. The conclusions of the authors are clear: the behaviour of physicians is still largely contrary to guidelines, suggesting that either cultural or marketing rather than legal factors are mainly responsible for morphinofobia. Staton [35] reports a significant difference in the perception of pain between physicians and patients, especially among certain ethnic groups (Afro-Americans). Nishimori [36] studying opiates abuse among patients at home reported treatment failure by physician in case of opioïde dependency. Ballantyne *et al. *[37] in a review of literature on chronic pain treatment with opioïdes shows discrimination in prescribing morphine in relation with fears of dependencies.

Many studies report fears and false beliefs concerning the use of morphine in pain management among HP [14,22,38-40]; e.g. Larue *et al. *[41] report false beliefs of general practitioners and oncologists in France; negative attitudes of nurses in the use of morphine in pain management are reported from Australia [22] from the USA [23] and from Hong Kong [42].

The existence of false beliefs on pain, addiction and abuse of morphine have also been reported by Gilson *et al. *[21] in a study among 300 American physicians. Furthermore Nwokeji *et al. *[43] reported that among 267 general practitioners who agreed to prescribe opioïdes to patients suffering from chronic non-cancerous pains, half feared addiction and abuse. White *et al. *[44] studying the attitudes of hospital physicians on opiate prescription, confirm that opiophobia is often related to fears of dependency. Devi *et al. *[45] questioning 253 Malaysian physicians reported that 83% of the respondents consider a possible addiction and the fear of exceeding sedation and respiratory depression as the main obstacles in prescribing morphine. Clinical documented experiences have proven that these fears are not justified [3,46-49]. Some physicians may also lack knowledge on morphine pharmacokinetics or may be unfamiliar with morphine prescription [50]. Ripamonti et al., [16] concluded in an Italian study of cancer patients that despite the WHO guidelines and EAPC recommendations, there was an inappropriate use of transdermal opioids by Italian physicians in situations where the use of oral morphine was not contraindicated.

Our results showed a rather weak relationship between **socio-demographic features **and the perceptions of the use of morphine in pain management. Yet morphinofobia was highest among little-educated older men living in rural areas. The cultural and geographic influences on attitudes and beliefs regarding morphine among patients with non cancerous pains have been stressed by Monsivais *et al. *[51] and Cicero *et al. *[52]. However a literature review by Turk [53] is cautious in this regard. Ripamonti et al., [16] mentioned that patients were having a problem in taking morphine but they had no cultural problems with other opioids. Most patients knew what morphine meant but do not know the role and the potency of other opioids.

Health professionals play an important role as far as morphinofobia is concerned, be it through a possible lack of knowledge regarding morphine [23,54], be it out of "more philosophical" reasons as suggested by Covington [10] and Bandieri et al., [13]. Yun *et al. *[55] and Edwards *et al. *[22] therefore suggest the necessity to develop more positive attitudes among HP regarding the use of morphine.

There are limits to our study. First a generalisation to the population of Beira Interior of our observations might not be indicated because of the small sample of GP and its opportunistic nature. Second, our study focused on attitudes and perceptions on morphine of GP (potential patient) and HP and did not take in consideration the patients' vision. Third, it should be kept in mind that some of our results are matter of debate in the specialized literature [25,52]. Last, our study concentrated on the concept of morphinofobia and should not be generalized for other opioids.

## Conclusion

This study contributes to a better understanding of "the myths of morphine" among the general population and health professionals in the region of Beira Interior. It suggests that efficient pain management is not limited to the prescription of an adequate analgesic according to « the golden standard ». The success of a morphine prescription is influenced by a multitude of other factors.

Our results are in accordance with the results of the study by Musi *et al*. [26] done in a similar regional setting. There seems to be quite a misunderstanding of "morphine" as well among GP as HP in North-Eastern Portugal. Such a misunderstanding might well end up in straight forward morphinofobia, thus ultimately compromising an appropriate pain management strategy as recommended by W.H.O. guidelines and EAPC recommendations.

This leads us to suggest that there is a need for information campaigns targeting the general population and for better training programs targeting health care professionals based on the theory of planned clinical behaviour [14] in order to improve acceptance and efficacy of pain management.

## Competing interests

The authors declare that they have no competing interests.

## Authors' contributions

HV carried out the study concept, drafted the manuscript, the data analysing and interpretation, the follow-up and participated in the questionnaire design and data collection. EKM carried out the design of the questionnaire, the study concept and participated in the draft of the manuscript. MF carried out the design and the translation of the questionnaires, survey and data collection and contributed in the data analysis and interpretation. CHR conceived the study, participated in the questionnaire design, data analysis and interpretation and the draft of the manuscript. PC carried out the draft of the manuscript and participated in the data analysis and interpretation. All the authors approved the final manuscript.

## Pre-publication history

The pre-publication history for this paper can be accessed here:

http://www.biomedcentral.com/1472-684X/9/15/prepub
